# New Progress on the Role of Glia in Iron Metabolism and Iron-Induced Degeneration of Dopamine Neurons in Parkinson’s Disease

**DOI:** 10.3389/fnmol.2017.00455

**Published:** 2018-01-19

**Authors:** Huamin Xu, Youcui Wang, Ning Song, Jun Wang, Hong Jiang, Junxia Xie

**Affiliations:** Collaborative Innovation Center for Brain Science, Department of Physiology, Shandong Provincial Collaborative Innovation Center for Neurodegenerative Disorders, Key Laboratory of Pathogenesis and Prevention of Neurological Disorders and State Key Disciplines: Physiology, Medical College of Qingdao University, Qingdao, China

**Keywords:** Parkinson’s disease, iron, glia, dopamine neurons, iron transporters

## Abstract

It is now increasingly appreciated that glial cells play a critical role in the regulation of iron homeostasis. Impairment of these properties might lead to dysfunction of iron metabolism and neurodegeneration of neurons. We have previously shown that dysfunction of glia could cause iron deposit and enhance iron-induced degeneration of dopamine (DA) neurons in Parkinson’s disease (PD). There also has been a substantial growth of knowledge regarding the iron metabolism of glia and their effects on iron accumulation and degeneration of DA neurons in PD in recent years. Here, we attempt to describe the role of iron metabolism of glia and the effect of glia on iron accumulation and degeneration of DA neurons in the substantia nigra of PD. This could provide evidence to reveal the mechanisms underlying nigral iron accumulation of DA neurons in PD and provide the basis for discovering new potential therapeutic targets for PD.

## Introduction

Parkinson’s disease (PD) is a common neurodegenerative disorder characterized by resting tremor, rigidity, and bradykinesia. Neuropathological hallmarks of PD include the degeneration and loss of dopaminergic neurons in the substantia nigra (SN) and the subsequent dopamine (DA) depletion in the striatum. Although the exact pathogenesis of PD is not fully understood, a growing body of research has confirmed that nigral iron accumulation was involved in the death of DA neurons in PD (Wang et al., 2007; Jiang et al., 2010, 2017; Song et al., 2010). Iron levels in the substantia nigra pars compacta (SNpc) increased significantly, while no significant change in the SN pars reticularis of PD patients (Dexter et al., 1989). Then many researchers have confirmed that iron levels in the SN were significantly higher in PD patients than normal subjects using a variety of technologies such as biochemistry, histochemistry, and imaging (Dexter et al., 1991; Sofic et al., 1991; Langkammer et al., 2016). About 90% of the patients with idiopathic PD showed an increased echogenicity of SN using transcranial sonography (TCS). Further experiments confirmed that there was a significant positive correlation between the echogenic area of the SN and the concentration of iron, H-ferritin and L-ferritin in post-mortem brains (Zecca et al., 2005; Berg, 2006). In recent years, using magnetic resonance imaging (MRI), susceptibility weighted imaging (SWI), enhanced gradient echo T2^∗^ weighted angiography (ESWAN), Quantitative susceptibility mapping (QSM) *in vivo* also confirmed increased nigral iron content in PD patients (Wang C. et al., 2013; Pyatigorskaya et al., 2014; Wu et al., 2014; Langkammer et al., 2016; Huddleston et al., 2017). In addition, results showed that iron levels in the SN were associated with the severity of motor symptoms in PD patients (Martin et al., 2008; Wallis et al., 2008; Pavese and Brooks, 2009; Guan et al., 2017).

Conventional MRI and diffusion-weighted imaging at 1.5 T have been recommended by European Federation of Neurological Societies (EFNS) to support a diagnosis of multiple system atrophy (MSA) or progressive supranuclear palsy versus PD (Berardelli et al., 2013). EFNS has also recommended TCS for the differentiation of PD from atypical and secondary parkinsonian disorders and for the early diagnosis of PD and in the detection of subjects at risk for PD. They also mentioned that TCS should be used in conjunction with other screening tests (Berardelli et al., 2013). However, it has been reported that the diagnostic accuracy of TCS in early stage PD is not sufficient for routine clinical use (Bouwmans et al., 2013). In their study, 196 consecutive patients were collected for analysis of clinically unclear parkinsonism by undergoing a TCS scan of the brain. Two years later, patients were re-examined for a final clinical diagnosis. Results showed that the sensitivity of TCS of SN+ for the diagnosis idiopathic Parkinson’s disease (IPD) was 0.40 and the specificity was 0.61. Therefore, it might not sufficient to use these techniques as a routine basis for potential PD patients before the symptoms. However, longer follow-up periods might probably increase diagnostic accuracy. More studies should be conducted to identify subjects in a pre-symptomatic phase of PD using these technologies in the future.

**FIGURE 1:**
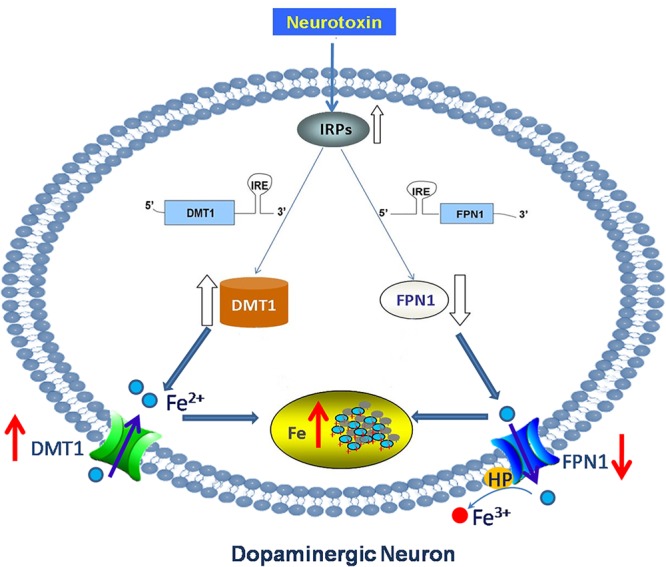
Abnormal expression of iron transporters caused iron accumulation in DA neurons in PD. Neurotoxin induced up-regulation of iron importer DMT1 and down-regulation of iron export protein FPN1 in PD via activation of IRPs. This abnormal expression of iron transporters caused iron accumulation and enhanced iron-induced neurotoxicity in dopaminergic neurons in PD.

It is also concluded that neurodegenerative diseases involving iron-mediated toxicity may be due to a failure of iron transport or storage mechanisms, rather than to the presence of high levels of non-transferrin-bound iron (NTBI) (Bishop et al., 2011). There are two kinds of iron transport processes in the brain: transferrin (Tf) binding iron (Tf-Fe) and NTBI. A list of abbreviations and the functions of iron-related proteins are shown in **Table 1**. Our previous study and others have confirmed that increased iron levels were associated with increased expression of iron importer divalent metal transporter 1 (DMT1) and decreased expression of iron exporter ferroportin1 (FPN1) in PD animal and cell models (Salazar et al., 2008; Wang et al., 2009; Jiang et al., 2010). The activation of iron regulatory proteins (IRPs) was responsible for this abnormal expression of iron transporters (Salazar et al., 2008; Wang et al., 2009; Jiang et al., 2010) (**Figure 1**). Increased iron and DMT1 expression were also observed in post-mortem PD patients (Salazar et al., 2008). This indicated that abnormal expression of iron transporters caused iron accumulation and enhanced iron-induced neurotoxicity in PD.

**Table 1:** A list of abbreviations and the functions of iron related proteins.

	Abbreviation	Function
Divalent metal transporter 1	DMT1	Cellular iron importer responsible for ferrous iron uptake
Ferroportin1	FPN1	Cellular iron exporter responsible for ferrous iron release
Ceruloplasmin	Cp	A ferroxidase mediating oxidization of Fe^2+^ to Fe^3+^ and promoting FPN1-mediated iron release
Transferrin/transferrin receptor 1	Tf/TfR1	A major pathway for Fe^3+^ ions acquisition to cells by a receptor-mediated pathway
Lactoferrin/lactoferrin receptor	Lf/LfR	Transfers Fe^3+^ ions to cells by a receptor-mediated pathway
Ferritin	Ferritin	Intracellular iron-storage protein, keeping iron in a non-toxic form
Hepcidin	Hepcidin	A circulating peptide that binds to FPN1, mediating degradation of FPN1

**FIGURE 2:**
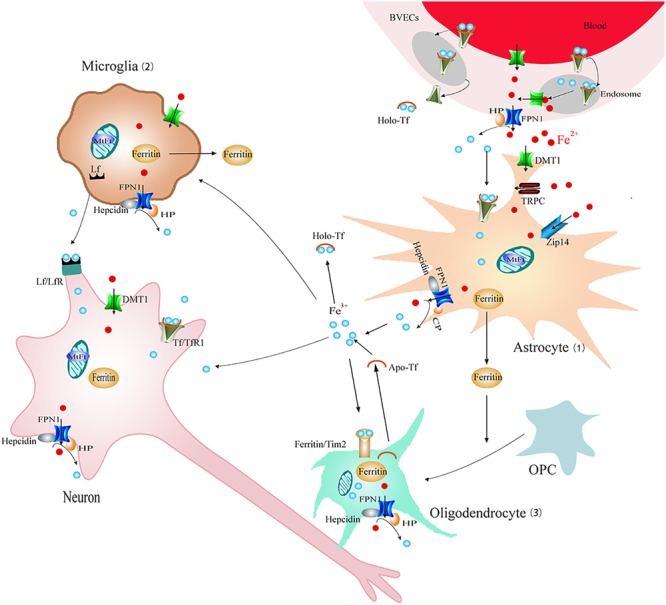
Schematic illustration of brain iron metabolism. Iron could cross BBB though endocytosis of holo-Tf followed by iron detached from Tf inside endosomes and FPN1-mediated iron efflux or transcytosis of holo-Tf through the BVECs. (1) Astrocyte: astrocytes could uptake Fe^3+^ via Tf-TfR1. DMT1, Zip14, and TRPC participate in Fe^2+^ absorption. Cp can oxidize Fe^2+^ to Fe^3+^ and then promote FPN1-mediated Fe^2+^ release. Iron can be stored in ferritin efficiently. (2) Microglia: Fe^2+^ could be transported via DMT1-mediated iron import and FPN1-mediated iron export. Microglia also can transfer Fe^3+^ ions to neurons by Lf/LfR-mediated pathway and store iron in ferritin. (3) Oligodendrocytes: iron is stored in oligodendrocytes mainly in the form of ferritin or Tf. Tf could be released from oligodendrocytes. Tim2-induced ferritin uptake is considered as the main mechanism for iron intake. Ferritin released from astrocyte and microglia promotes OPC maturation. BBB, blood–brain barrier; BVECs, brain capillary endothelial cells; Cp, ceruloplasmin; FPN1, ferroportin1; Lf/LfR, lactoferrin/lactoferrin receptor; NTBI, non-transferrin-bound iron; OPC, oligodendrocyte precursor cell; Tf/TfR1, transferrin/transferrin receptor 1; TRPC, resident transient receptor potential channel; Zip14, Zrt/Irt-like protein 14.

Furthermore, it is now increasingly appreciated that glia might be critically involved in the pathophysiology of PD. Glia are mainly classified as astrocytes, microglia, and oligodendrocytes. Both activation of astrocytes and microglia are found in the SN of PD (Shin et al., 2015). Activated astrocytes and microglia could remove damaged cells and protect neurons by releasing neurotrophic factors. Alternatively, they can also mediate neuron injury by releasing proinflammatory factors which may be involved in neuronal degeneration. Recently, attention has been drawn to the new insights into the function of glia. It is now increasingly appreciated that glia also play a critical role in the regulation of iron homeostasis and impairment of these properties might lead to dysfunction of iron metabolism and degeneration of DA neurons in PD. Astrocytes, microglia, and oligodendrocytes are all equipped with different iron-related proteins responsible for iron uptake, storage, use and export (**Figure 2**). In addition, cultured neurons, astrocytes and microglia all have the ability to store huge amounts of iron, but compared to neurons, glia can stored iron more effectively (Bishop et al., 2011). Among them microglia were the most efficient in NTBI accumulation (Bishop et al., 2011). Furthermore, astrocytes were involved in the formation of blood–brain barrier (BBB). About 95% of the capillary surface is covered by end feet of astrocytes (Dringen et al., 2007). Therefore, astrocytes are of vital importance for iron transport across BBB and maintain brain iron homeostasis (Dringen et al., 2007). This might be the main source of iron for neurons and microglia (**Figure 2**). In addition, studies found that iron overload could activate microglia and astrocytes and promote the release inflammatory factor and neurotrophic factors, which were involved in the regulation of iron metabolism of DA neurons (Wang J. et al., 2013; Zhang H.Y. et al., 2014).

Therefore, in this review, we describe the involvement of glia in pathophysiology of PD. Then we summarize iron metabolism of glia and the effect of glia on nigral iron accumulation and degeneration of DA neurons in PD. This could provide evidence to reveal the mechanisms underlying the effect of glia on iron accumulation of DA neurons in PD and provide the basis for discovering new potential therapeutic targets for PD.

## Effect of Microglia on Iron Accumulation and Degeneration of DA Neurons in PD

### Activation of Microglia in PD

Microglia are considered as resident macrophages in the brain where they participate in phagocytosis, immune surveillance, and neuroinflammatory processes. Although the etiology of PD is not yet elucidated, increasing evidence implicates that microglia-mediated inflammatory processes contribute to the degeneration of DA neurons in PD (Ransohoff, 2016). Results also showed that the activation state of microglia rather than the number of microglia in the SN contributed to microglia-induced neurotoxicity of DA neurons (Shin et al., 2015). The role of activated microglia in PD has been well described in previous reports. Post-mortem brain examination results showed that there were many activated microglia in the brain of PD patients. And activated microglia were mainly distributed in the SN where degeneration of DA neurons occurred (Banati et al., 1998). This might be mainly due to the highest density distribution of microglia in the normal SN area of the brain (Beach et al., 2007). It has been proposed previously that mutated α-synuclein could activate microglia with proinflammatory response (Su et al., 2009). This process occurred even before nigral neuronal loss in the SNpc (Bishop et al., 2011). These observations in PD patients support the presumption that activation of microglia is involved in the initiation and progression of PD (Bruck et al., 2016). Although it is not clear whether microglia activation is a causal or the result of the secondary event in PD, microglia activation-mediated inflammatory processes indeed lead to a vicious circle between inflammatory reaction and neuron damage, and this aggravates the symptoms of PD (Block and Hong, 2007; Neher et al., 2011).

On the other hand, activated microglia could also participate in neuroprotection (Le et al., 2016). This “double-edged sword” effect of microglia might depend on different activation states of microglia response to different types of stimuli in normal and disease conditions. It is now recognized that there exist two different activation states of microglia (Colton, 2009; Cherry et al., 2014). One is classical activation (M1 phenotype), which contributed to the inflammatory response to produce inflammatory cytokines. This is necessary for antigen presentation to kill intracellular pathogens. However, constant production of inflammatory cytokines could induce cell death in disease conditions. The other state is alternative activation (M2 phenotype), which had an anti-inflammatory phenotype responsible for repair and debris clearance. The proper transition from the M1 to M2 phenotype might be critical for microglia to efficiently end the inflammatory response. However, in PD conditions, persistent released inflammatory cytokines by microglia in the SN usually overshadow the beneficial molecules. It has been hypothesized that lack of M2 phenotype might be an important mechanism involved in neurodegeneration (Cherry et al., 2014).

The classical view considers that neurons are just passive victims of the activation of microglia, but in fact, neurons are not merely passive victims (Biber et al., 2007). It is now widely accepted that the interaction between neurons and glia together maintains tissue homeostasis in the central nervous system (CNS). The cluster of differentiation 200 (CD200), belonging to the immunoglobulin superfamily, participates in the regulation of immune response. CD200 is mainly expressed in neurons, which can act on CD200 receptor (CD200R) in microglia and maintain the microglia in the resting state (Lyons et al., 2007). Impairment of CD200-CD200R pathway induced activation of microglia in the SN and thus participated in the degeneration of DA neurons in PD (Wang et al., 2011).

### Microglia in Iron Accumulation and Degeneration of DA Neurons in PD

The prominent hallmarks of neuroinflammation are microglia activation and subsequent secretion of pro-inflammatory cytokines such as interleukin-1β (IL-1β) and tumor necrosis factor-α (TNF-α) (Mogi et al., 1994, 1996). Elevated release of IL-1β and TNF-α from activation of microglia was observed in the cerebrospinal fluid, as well as SN and striatum in post-mortem brain of PD patients (Mogi et al., 1994, 1996). And directly injection of IL-1β and TNF-α into the brain tissue can induce the degeneration of DA neurons (Carvey et al., 2005). It has been reported that TNF-α and transforming growth factor beta 1 (TGF-β1) could up-regulate iron import protein DMT1 and down-regulate iron export protein FPN1 in microglia. This increased iron uptake and decreased iron efflux promoted iron accumulation in microglia (Rathore et al., 2012). This accumulated iron in microglia might decrease extracellular iron levels, thus protect DA neurons against iron-induced neurotoxicity in the brain. However, studies have also shown that microglia activation might participate in iron-induced dopaminergic neurodegeneration in the SN (Zhang W. et al., 2014). Their results showed that Fe^2+^-induced loss of DA neurons was more severe in rat neuron–microglia–astroglia cultures than that in neuron–astroglia cultures, indicating the pivotal role of microglia in iron-elicited dopaminergic neurotoxicity. The mechanism is associated with activation of nicotinamide adenine dinucleotide phosphate oxidase 2 (NOX_2_) in microglia, thus producing many immune inflammatory factors (Zhang W. et al., 2014). They further confirmed that NOX_2_^-/-^ mice were resistant to iron-induced neurotoxicity in DA neurons, indicating that iron-elicited dopaminergic neurotoxicity is dependent on NOX_2_ activation of microglia. Therefore, inhibiting excessive activation of NOX_2_ in microglia may be new targets for the treatment of PD.

**FIGURE 3:**
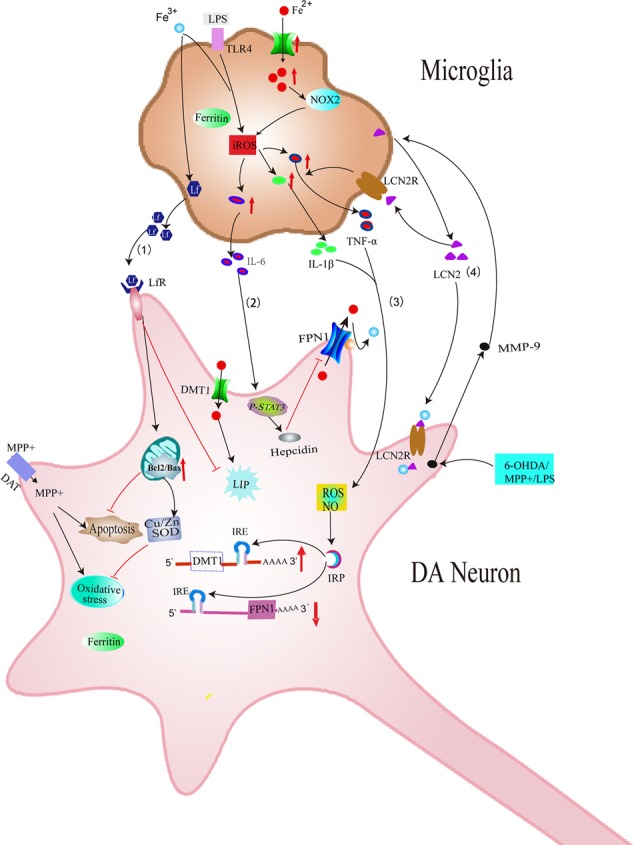
Schematic illustration showing several effects of microglia on iron metabolism and degeneration of DA neurons in PD. (1) Iron overload-induced activation of microglia increases the release of Lf, which provides neuroprotection against MPP^+^ in DA neurons. (2) LPS could induce the release of IL-6 in microglia, which could increase the expression of hepcidin in DA neurons, thus inhibiting FPN1-mediated iron efflux in DA neurons. (3) Excessive activation of microglia induced by LPS or neurotoxins could release IL-1β and TNF-α, which aggravates iron accumulation of DA neurons by up-regulating DMT1+IRE and down-regulating FPN1. (4) MMP-9 released by damaged DA neurons could lead to upregulation and release of LCN2. LCN2 then activates microglia to release TNF-α and IL-1β, which is involved in the abnormal expression of DMT1 and FPN1 in DA neurons. LCN2 released from activated microglia might also induce direct neurotoxicity via excessive iron delivery into DA neurons by binding to LCN2 receptors.

In addition, our previous study showed that iron status of microglia can also affect secretion of pro-inflammatory cytokines including IL-1β and TNF-α and then participate in the degeneration of DA neurons (Wang J. et al., 2013). Our results demonstrated that lipopolysaccharides (LPS) could activate microglia, resulting in abundant IL-1β and TNF-α secretion. This is enhanced by iron repletion and attenuated by iron depletion. This provides evidence that iron status of microglia is vital important for IL-1β and TNF-α releasing from microglia. Furthermore, IL-1β and TNF-α released from microglia also could affect iron metabolisms of DA neurons. Our previous study showed that IL-1β and TNF-α induced activation of IRP1, thus up-regulated DMT1 with iron responsive element (DMT1+IRE) expression and down-regulated FPN1 expression in ventral mesencephalon (VM) neurons (Wang J. et al., 2013). This is responsible for the increased iron influx and decreased iron efflux of VM neurons, thus leading to iron load of DA neurons. This led to a hypothesis that excess iron in the SN area activated microglia and released proinflammatory factors, thus aggravating iron accumulation inside DA neurons (**Figure 3**).

**FIGURE 4:**
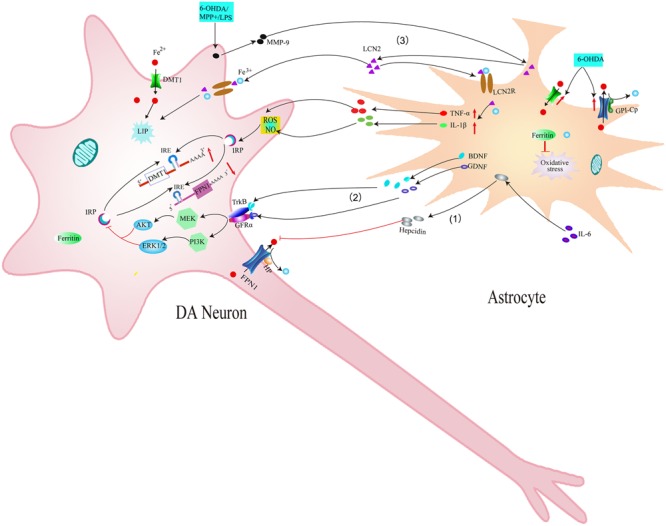
Schematic illustration showing the effects of astrocytes on iron accumulation in dopaminergic neurons in PD. (1) IL-6 could promote astrocytes to release hepcidin, which then prevents FPN1-mediated iron release from DA neurons. (2) BDNF and GDNF secreted by activated astrocytes can inhibit IRP via acting on their receptors, thus down-regulating the expression of DMT1 and reducing iron accumulation in DA neurons. (3) MMP-9 released by damaged DA neurons could lead to upregulation and release of LCN2. LCN2 then activates astrocytes to release TNF-α and IL-1β, which up-regulates DMT1 and down-regulates FPN1 in DA neurons. Released LCN2 might also induce direct neurotoxicity via excessive iron delivery into DA neurons by binding to LCN2 receptors.

Another possible mechanism underlying the effect of microglia on iron metabolism of neurons is associated with interleukin-6 (IL-6). Studies have found that LPS could induce the expression and release of IL-6 in microglia. Released IL-6 from microglia could up-regulate hepcidin via IL-6/signal transducer and activator of transcription 3 (STAT3) signaling pathway in neurons (Qian et al., 2014). Hepcidin is a critical regulator of the entry of iron into cells by binding to the iron exporter FPN1 and resulting in the internalization of FPN1, which could inhibit iron export from neurons. Therefore, IL-6 released by activated microglia up-regulated hepcidin of neurons and enhanced iron accumulation by preventing FPN1-mediated iron release from neurons (**Figure 3**). A recent result showed that activated microglia could also stimulate astrocytes to release hepcidin via IL-6 signaling, which then prevented FPN1-mediated iron release and induced apoptosis of neurons (You et al., 2017) (**Figure 4**). In addition, ceruloplasmin (Cp) is one of the major copper-binding proteins responsible for converting toxic ferrous iron into ferric iron (Patel and David, 1997). Evidence has shown that Cp could potentiate LPS-induced activation of microglia and increase the production of IL-6 (Lazzaro et al., 2014). These findings provide powerful evidence that the cooperative effect of neuroinflammation and iron accumulation may enhance the degeneration of DA neurons in PD.

In addition to release TNF-α, IL-1β, activated microglia can also release lactoferrin (Lf), which is an iron-binding protein belonging to the transferrin family. Lf-Lf receptor (LfR) transfers Fe^3+^ ions by a receptor-mediated pathway. Iron affinity of Lf is about 300 times higher than TfR (Baker et al., 1994). It was reported that both iron-free Lf (apo-Lf) and iron-saturated Lf (holo-Lf) entered Caco-2 cells via a similar mechanism, but affect cell proliferation differentially (Jiang et al., 2011). In the brain, Lf is produced by activated microglia (Fillebeen et al., 2001). The expression of Lf mRNA was reported to be increased in 1-methyl- 4-phenyl-1,2,3,6-tetrahydropyridine (MPTP) mouse models of PD (Faucheux et al., 1995). Immunohistochemical studies of PD patients revealed an increase of LfR on SNpc neurons and microvessels (Faucheux et al., 1995). These findings indicate a possible role of Lf/LfR in nigral iron accumulation and the subsequent degeneration of dopaminergic neurons in PD. Our previous study suggested that activated microglia could synthesize and release Lf. This process was further enhanced by iron overload (Wang et al., 2015). In VM neurons, both apo-Lf and holo-Lf exerted their neuroprotective effects against 1-methyl-4-phenylpyridine (MPP^+^) by protecting mitochondria, increasing the expression of copper and zinc-containing superoxide dismutase (Cu/Zn-SOD) and B-cell lymphoma-2 (Bcl-2) (Wang et al., 2015). This indicated that Lf protected dopaminergic neurons from neurotoxin, although Lf tended to transport iron to dopaminergic neurons. This might be related to the anti-oxidant and anti-apoptotic activities of apo-Lf and holo-Lf. Chelation of cellular iron by apo-Lf might also exert its function via decreasing cellular free iron and iron-induced neurotoxicity (Wang et al., 2015) (**Figure 3**).

## Effect of Astrocytes on Iron Accumulation and Degeneration of DA Neurons in PD

### Activation of Astrocytes in PD

Astrocytes are the most abundant cell type in the CNS and have diverse physiological functions including cellular support during CNS development, ion homeostasis, uptake of neurotransmitters and neuromodulation through their close association and communication with neurons and other glia. There is abundant evidence for a protective effect of astrocytes on the survival of DA neurons. Intranigral infusion of IL-1-β to activate astrocytes in advance can effectively protect DA neurons from 6-hydroxydopamine (6-OHDA)-induced neurotoxicity (Saura et al., 2003). They mentioned that in this condition, microglial activation was not induced by IL-1β. This was a key factor for the neuroprotection as activated microglia is potentially neurotoxic to DA neurons. They suggested that the protective effects in IL-1β-treated animals were associated with activated astrocytes, but not a direct effect of the IL-1β (Saura et al., 2003). Our previous study showed that activation of heme oxygenase-1 (HO-1) in astrocytes might responsible for the protective effect of astrocytes on DA neurons by resisting oxidative stress in the MPTP-induced PD mice models (Xu et al., 2016).

It has been accepted that astrocytes have both neuroprotective and neurodegenerative functions. Whether astrocytes are beneficial or harmful might depend largely on the molecules that they release into and uptake from the extracellular space (Rappold and Tieu, 2010). It is well documented that nerve growth factor (NGF), glial cell line-derived neurotrophic factor (GDNF), and basic fibroblast growth factor (bFGF) released from astrocytes promote the survival of DA neurons (Rappold and Tieu, 2010; Rocha et al., 2012). Astrocytes might also confer neuroprotection to DA neurons by clearing excess extracellular toxic alpha-synuclein and enhancing degradation of alpha-synuclein through the lysosomal pathway. However, although this degradation of alpha-synuclein in astrocytes may confer initial protection to neurons, when the accumulation of alpha-synuclein exceeds the degradation capacity of astrocytes, aggregates of alpha-synuclein in astrocytes could up-regulate transcripts of inflammatory cytokines such as IL-1β, and TNF-α (Lee et al., 2010; Lindstrom et al., 2017). It has been found that α-synuclein-positive protein aggregates were present in the astrocytes of post-mortem PD brains (Wakabayashi et al., 2000). A study on α-synuclein inducible transgenic mice, which selectively expressed human PD-related A53T α-*synuclein* in astrocytes, showed that excess A53T α-synuclein in astrocytes caused severe astrogliosis, leading to dysfunction of astrocytes to maintain the integrity of BBB and homeostasis of extracellular glutamate. This induced inflammation, microglial activation and a significant loss of DA neurons in the midbrain in these mutant mice (Gu et al., 2010).

In addition, activated astrocytes also possess immune and inflammatory activities just as microglia. This also called reactive astrogliosis, which is accompanied by neuronal injury in neurodegenerative conditions including PD. This process could limit damage within the reaction area and provide repairment after injury (Biber et al., 2007; Pyatigorskaya et al., 2014). However, studies have shown that there might be two different types of reactive astrogliosis depended on the type of inducing injury. Neuroinflammation and ischemia induced two different types of reactive astrocytes termed “A1” and “A2,” respectively. Reactive astrocytes in ischemia exhibited a phenotype that might be beneficial or protective (A2), whereas reactive astrocytes induced by LPS exhibited a phenotype that might be detrimental (A1) (Zamanian et al., 2012; Lindstrom et al., 2017). Recently, it has been reported that A1 reactive astrocytes could be induced by IL-1β and TNF-α secreted by activated neuroinflammatory microglia. These A1 reactive astrocytes lost most their normal functions, but gained a new neurotoxic function, which induced the death of neurons and oligodendrocytes (Liddelow et al., 2017). This indicated that activated neuroinflammatory microglia could induce A1 reactive astrocytes by releasing TNF-α and IL-1β. A1 reactive astrocytes then amplified the immune response and ultimately contributed to the cell death of DA neurons in the SNpc during neurodegeneration (Saijo et al., 2009; Glass et al., 2010).

### Iron Metabolism in Astrocytes and Its Role in Degeneration of DA Neurons

Astrocytes participate in the formation of BBB and are generally accepted as principle contributor for the uptake of a variety of nutrients including iron to the brain. It controls the process of iron transport from outside the brain to inside the brain and regulates iron transport from astrocytes to other brain cells (Dringen et al., 2007). Studies have shown that astrocytes are not cells with a high metabolic requirement for iron. Iron content in basic condition was only about 10 nmol/mg protein (Hoepken et al., 2004; Riemer et al., 2004). However, these cells have a strong iron transport capacity and can transport Tf-Fe, NTBI and heme iron. Studies have shown that astrocytes *in vivo* do not express Tf or TfR1 (Moos, 1996). However, these two proteins were expressed in cultured astrocytes *in vitro* to participate in iron transportation (Hoepken et al., 2004). Most studies suggest that astrocytes do not give priority to uptake Tf-Fe *in vivo* or *in vitro* (Swaiman and Machen, 1985; Oshiro et al., 1998; Takeda et al., 1998; Jeong and David, 2003). DMT1 is thought to participate in divalent iron absorption in astrocytes (Tulpule et al., 2010). DMT1 was detected in cultured astrocytes (Jeong and David, 2003; Erikson and Aschner, 2006) and mainly expressed in the end-foot which related to the vascular endothelial cells (Burdo et al., 2001; Wang et al., 2001), indicating a major role of DMT1 in astrocytes for brain iron uptake. Release of iron from vascular endothelial cells was uptaken by nearby astrocytes via DMT1 and then redistributed to other cells. This suggests that DMT1 might be involved in the redistribution of iron in the brain. In addition, it has been found that the zinc transporter Zip14 (Bishop et al., 2010) and resident transient receptor potential channel (TRPC) (Pelizzoni et al., 2013) in astrocytes also play a role in the process of NTBI transportation (**Figure 2**). Iron absorption mediated by these transporters in astrocytes can buffer high levels of extracellular iron, and thus inhibit high iron-induced damage to DA neurons. In addition, astrocytes can store iron via ferritin efficiently and release iron by FPN1. Cp is a kind of ferrous oxidase mainly in the form of glycosyl-phosphatidylinositol (GPI)-Cp in the brain (Patel and David, 1997) and can effectively oxidize Fe^2+^ to Fe^3+^, then promoting FPN1-mediated iron release (**Figure 2**). FPN1 and GPI-Cp co-expressed in the cell surface of astrocytes (Jeong and David, 2003) to mediate iron release from astrocytes (**Figure 2**).

It has been reported that iron levels can affect the expression of iron related proteins in astrocytes. Ferric iron incubation increased ferritin expression and reduced the expression of TfR (Hoepken et al., 2004). This adjustment is beneficial to reduce iron uptake and decrease intracellular free iron levels in the high iron environment. This could protect astrocytes against iron-mediated oxidative stress. Our previous study confirmed that regulatory mechanism of iron metabolism in astrocytes was significantly different from DA neurons after 6-OHDA treatment. 6-OHDA induced an increase in DMT1-mediated ferrous iron influx and a decrease in FPN1-mediated iron outflow, then led to iron accumulation in DA neurons (Wang et al., 2009; Jiang et al., 2010). However, in astrocytes, both iron import and export were enhanced by 6-OHDA due to the significantly increased expression of DMT1 and FPN1 (Zhang et al., 2013). This suggested that 6-OHDA might promote iron transport rate in astrocytes under the condition of oxidative stress to avoid iron deposition in astrocytes.

Astrocytes may affect the iron metabolism of DA neurons in PD models. Astrocytes are vital for the survival of DA neurons by secreting various neurotrophic factors, such as brain derived neurotrophic factor (BDNF) and GDNF. Lui et al. (2012) observed higher expression of BDNF in the damaged striatum and SN and elevated expression of GDNF in the damaged striatum in early 6-OHDA-induced PD models (5 and 7 days after unilateral injection of 6-OHDA); Both of GDNF and BDNF decreased after 6-OHDA treatment for 14 days. This indicated that synthesis and secretion of BDNF and GDNF by astrocytes in the early PD rat models can promote cell survival (Lui et al., 2012). Our previous study demonstrated that BDNF and GDNF can inhibit iron uptake into neurons by decreasing the expression of iron import protein DMT1, thus reducing 6-OHDA-induced iron accumulation in DA neurons. Intracellular signaling pathways MEK/ERK, PI3K/Akt might participate in these processes (Zhang H.Y. et al., 2014) (**Figure 4**). These results confirmed that astrocytes can affect iron metabolism and survival of neurons by releasing neurotrophic factors BDNF and GDNF.

Recently, another novel mechanism underlying neuron-glia interaction in iron metabolism has been reported (Kim et al., 2016). Lipocalin-2 (LCN2) is a member of highly heterogeneous secretory protein family of lipocalin. Diverse functions of lipocalin-2 have been demonstrated in the CNS (Ferreira et al., 2015; Jha et al., 2015). It has been reported that LCN2 was up-regulated in the SN of PD patients and MPTP-induced PD animal models (Kim et al., 2016). Further study showed that the increased LCN2 levels contributed to neurotoxicity and neuroinflammation, resulting in disruption of the nigrostriatal DA neurons and abnormal locomotor behaviors (Kim et al., 2016). Secreted LCN2 can activate microglia and astrocytes to promote M1 polarization and suppress M2 signaling pathway (IL-4-STAT6 signaling pathway) (Jang et al., 2013a,b; Lee et al., 2015). These activated astrocytes and microglia can produce neurotoxic cytokines such as TNF-α and IL-1β (Kim et al., 2016), which might be involved in the dysfunction of iron transporters and thus increase iron accumulation in DA neurons as mentioned above. In addition, LCN2 was reported to be an iron transport protein, regulating intracellular iron levels by binding to its receptor (Lee et al., 2012; Jha et al., 2015). Therefore, it is possible that increased secretion of LCN2 in reactive astrocytes and activated microglia might induce direct neurotoxicity to DA neurons via excessive iron delivery into DA neurons, resulting in the disruption of the DA neurons in PD (Kim et al., 2016) (**Figures 3, 4**). This provides new experimental evidence on relationship between abnormal iron metabolism and inflammatory in PD. Further studies should be conducted to elucidate the exact mechanisms underlying the effect of LCN2 on iron accumulation in DA neurons in PD.

## Iron Metabolism In Oligodendrocytes

Oligodendrocytes play a key role in myelin formation for proper transmission of nerve impulse in the CNS. There are large amounts of stored iron and synthesized Tf in oligodendrocytes (Todorich et al., 2009; Franco et al., 2015) (**Figure 2**). It has been shown that iron uptake of oligodendrocytes was accompanied by myelin formation and iron deficiency animals showed damage of myelin formation (Badaracco et al., 2008). In addition, injection of apotransferrin (aTf) to postnatal day 2–5 rats increased the expression of several myelin proteins and accelerated oligodendrocyte maturation (Escobar Cabrera et al., 1994, 1997; Marta et al., 2003). Transgenic mice with Tf overexpression also showed increased myelin formation (Saleh et al., 2003). These results indicate that iron and Tf were necessary molecules for myelin formation and maturation of oligodendrocytes (Franco et al., 2015).

Recently, results showed that hypomyelination in iron deficiency animals might be also associated with the deficiencies in microglia and astrocytes (Rosato-Siri et al., 2017). During postnatal development, microglia were an important iron source for oligodendrocytes (Todorich et al., 2009). There were large amounts of accumulated iron in microglia before myelination. However, iron levels decreased in microglia paralleled by increased iron accumulation in oligodendrocytes. This suggests that accumulated iron in microglia might be released from microglia to developing oligodendrocyte precursor cell (OPC) for its maturation during myelination (Todorich et al., 2009) (**Figure 2**). It has been demonstrated that microglia-released ferritin is an important source of iron for oligodendrocytes (Zhang et al., 2006). Further *in vivo* study showed that microinjection of ferritin to the spinal cord of adult rats could lead to internalization of ferritin in microglia and then ferritin could be subsequently released to promote the proliferation of neuron-glial antigen 2 (NG2)-positive progenitor cells and differentiation into mature oligodendrocytes (Schonberg et al., 2012). This indicates that ferritin released from microglia might act as a source of iron for NG2+ progenitor cells, thereby contributing to the proliferation and the formation of new myelin-producing oligodendrocytes. Astrocyte could also influence maturation and differentiation of oligodendrocytes through the secretion of different growth factors. In addition, iron efflux from astrocytes could be involved in remyelination of OPC directly (Schulz et al., 2012). In iron deficiency conditions, a crosstalk between astrocyte, microglia, and OPC prevented oligodendrocytes maturation and myelin formation.

Oligodendrocytes are the main iron-containing cells in the brain (Gerber and Connor, 1989). Iron was stored in oligodendrocytes mainly in the form of ferritin or Tf (**Figure 2**). Studies have shown that there were ferritin binding sites in oligodendrocytes (Hulet et al., 1999), indicating receptor- mediated mechanisms of iron transport (Fisher et al., 2007). It has been confirmed that T cell immunoglobulin and mucin domain-containing protein-2 (Tim2) is the receptor of heavy chain ferritin (H-ferritin). It can bind and result in internalization H-ferritin (Chen et al., 2005). Studies have confirmed the expression of Tim2 in oligodendrocytes *in vivo* and *in vitro*. As oligodendrocytes neither express TfR nor express DMT1 (Todorich et al., 2008). Tim2 is considered as the main mechanism for iron intake in oligodendrocytes (Todorich et al., 2008) (**Figure 2**). Another study showed that scavenger receptor class 5 (scara5) was the receptor of light chain ferritin (L-ferritin) expressed in embryonic mice and kidney cells of adult mice. Scara5 can bind to L-ferritin or Tf (but not HFt) and mediate its endocytosis (Li et al., 2009). The discovery of receptor-mediated iron transport in oligodendrocytes provides the new experimental basis for mechanisms of iron transportation and indicates its possible role in neurodegenerative diseases including PD. It is now considered that oligodendrocytes may not play a key role in the occurrence and development of PD, they may be more involved in the progression of late onset of PD (Halliday and Stevens, 2011).

## Conclusion and Future Directions

In recent years, considerable advances have been made in understanding iron metabolism in glia and neurons. We have summarized iron metabolism in glia (**Figure 2**) and reviewed their possible roles in the degeneration of DA neurons in PD (**Figures 3, 4**) in this review. Glia could affect iron metabolism and survival of DA neurons in PD through the release of proinflammatory factors or neurotrophic factors. Excessive activation of glia aggravated iron accumulation and degeneration of DA neurons in PD. In addition, there exists a complex regulatory mechanism between glia, which leads to the final degeneration of DA neurons. Therefore, regulating the function of glia may provide a new therapeutic target for the treatment of iron-mediated neurodegenerative disorders especially PD.

However, the current researches on iron metabolism rely mainly on experimental animals, especially in rodent models. These models cannot fully simulate changes of iron metabolism in the brain of PD patients. Therefore, it is crucial for studies using human stem cells, human glia or post-mortem brain tissue of PD patients to clarify iron metabolism of glia and their role in the degeneration of DA neurons in PD. Further investigations are also required to investigate whether the newly discovered transporters and regulatory proteins are also expressed in glia and how they functioned. In addition, the exact molecular and cellular mechanisms underlying the interaction between glia and DA neurons on iron metabolism should also be elucidated in the future.

## Author Contributions

HX wrote the manuscript. YW and HX constructed the figures. NS and JW contributed to the editing of the manuscript. HJ and JX revised the manuscript. All authors read and approved the final manuscript.

## Conflict of Interest Statement

The authors declare that the research was conducted in the absence of any commercial or financial relationships that could be construed as a potential conflict of interest.
